# Supporting fluid teams: a research agenda

**DOI:** 10.3389/fpsyg.2024.1327885

**Published:** 2024-01-25

**Authors:** Tripp Driskell, Gregory Funke, Michael T. Tolston, August Capiola, James Driskell

**Affiliations:** ^1^Florida Maxima Corporation, Orlando, FL, United States; ^2^Air Force Research Laboratory, Wright-Patterson Air Force Base, Dayton, OH, United States

**Keywords:** fluid teams, team performance, teamwork, group dynamics, team research

## Abstract

Fluid teams are teams that are rapidly assembled from across disciplines or areas of expertise to address a near-term problem. They are typically composed of individuals who have no prior familiarity with one another, who as a team must begin work immediately, and who disband at the completion of the task. Prior research has noted the challenges posed by this unique type of team context. To date, fluid teams have been understudied, yet their relevance and application in the modern workplace is expanding. This Perspective article presents a concise overview of critical research gaps and opportunities to support selection, training, and workplace design for fluid teams.

## Introduction

Temporary teams have been studied over the years in various guises as *ad hoc* teams (Lorge et al., 1958), emergent response teams (Majchrzak et al., 2007), and swift starting action teams (McKinney et al., 2005). Driskell et al. (2024) describe one specific type of temporary team as a *fluid team* comprised of four core characteristics; (1) team members are rapidly assembled to address an immediate problem, (2) members are assembled based on domain expertise and typically have no prior history or experience working together, (3) the team must begin work on a task that is immediate, time-critical, and of short duration, and (4) at completion of the task, the team disbands with little likelihood of further interaction. Such teams have become prevalent in various contexts such as healthcare (Bell et al., 2023, this issue; Grossman et al., 2024, this issue), innovation teams in industry (Linhardt and Salas, 2023, this issue), and the military (Capiola et al., 2020).

Fluid teams differ from traditional teams in a number of ways. Because they are rapidly assembled often from across disciplines, team members lack familiarity with others in the team. Because of the immediate nature of the task requirement, they have little time to orient themselves to other team members. Stemming from the short time frame of the team’s interaction, which may typically last from several hours to several days, these teams do not have the opportunity to develop properties such as cohesion or well-developed shared mental models gradually over time. Further, the team dissolves upon task completion with no anticipation of future interaction. These conditions pose unique challenges to effective team performance. In the following, we present a research agenda describing selection, design, and training approaches to support effective fluid team performance.

## Selection

Driskell et al. (n.d.) reviewed the team composition literature to shed light on composition considerations for forming fluid teams. Team composition research is concerned with identifying and arranging the sets of attributes present among team members that facilitate effective team performance. Whereas important implications can be drawn from the composition literature, the authors found that concrete conclusions regarding the effects of attribute composition on fluid team processes and performance were difficult to establish. Because fluid team members are assembled on short notice to address an immediate task, it is likely that the primary criteria for team member selection will be the specific technical competencies required for the task. However, it is important to further consider how the team is composed in terms of other attributes that can impact fluid team performance. This knowledge can support the development of team selection tools or technology platforms for composing fluid teams.

### Which team composition attributes are most influential in fluid team environments?

Research distinguishes between surface-level attributes of team members, which include overt easily distinguishable cues such as age or gender that are readily perceptible in short-term interactions, and deep level attributes such as values or personality which tend to manifest themselves over time (Driskell et al., 2006; Bell, 2007). We expect surface-level attributes to be most impactful in fluid teams in that characteristics such as age, gender, expertise, and occupational status or rank provide easily distinguishable cues to differentiate among team members in the short duration within which a fluid team initiates interaction. These cues provide a basis for team members to develop task expectations for other team members with whom they have no familiarity, and constitutes an initial orientation stage that structures team interaction (e.g., who is perceived to be competent, who’s ideas are most valuable, and so on). Research is needed to illuminate this process in fluid teams and document advantages (e.g., allowing swift interaction) and disadvantages (e.g., potential biases stemming from stereotype-based interaction).

The composition literature has also validated the importance of deep-level attributes on team performance. It is likely that deep-level attributes are more difficult to discern than surface-level attributes in time-compressed fluid team interaction. For example, team member personality is deemed to be an important factor in team functioning and performance (e.g., Driskell et al., 1987), but it is likely to be difficult to infer personality characteristics during limited fluid team interaction. On the other hand, some characteristics such as conscientiousness may be easily construed in a task environment where proxy cues such as hard-working versus social loafing behavior are readily apparent. Furthermore, deep-level values may be inferred from surface-level characteristics such as organizational membership. Further research is needed to examine the relative impact of various surface-level and deep-level cues in fluid teams, and more broadly, how rapidly formed fluid team members orient themselves to one another when they have neither initial familiarity nor the time to develop familiarity prior to task engagement.

Other potential research questions include:

- What is the value of existing team selection inventories for fluid team contexts (e.g., Burch and Anderson, 2004; Mathieu et al., 2015)?
- How do multiple attributes interact in fluid teams to impact team processes and performance? For example, an individual who is high on conscientiousness and low on sociability may be difficult to work with in a traditional team, but less so in the time-compressed, task-focused fluid team context.
- This question is also relevant at the group level of analysis. For example, in typical studies that investigate the relationship between age and gender composition and performance, age *or* gender diversity is used to predict performance, not age *and* gender diversity. That is, the distribution of multiple attributes is not often considered simultaneously (i.e., assessing trait interaction). Recent work by Emich et al. (2022) has begun to address this issue and further research is warranted.
- What lower-level facets are predictive in fluid team contexts? Many deep-level attributes are multifaceted; for example, the broad personality trait of extraversion has been viewed by some researchers as comprised of the lower level facets of sociability/affiliation and outgoing/assertiveness (Driskell and Salas, 2013). These facets may be differentially predictive of performance in fluid teams. That is, team member sociability may be less predictive of team outcomes in a fluid team context in which there is little opportunity for off-task interaction, whereas assertiveness may be predictive, and this relationship would be masked at a higher level of trait analysis.
- The presence of curvilinear effect relationships. Much of the composition literature has assumed linear relationships between composition attributes and team performance. This assumption has been called into question. For example, moderate levels of extraversion may benefit team performance, while too much or too little extraversion can be detrimental (see Driskell et al., 1987). In fluid teams, low to medium mean neuroticism may not impair performance, whereas high neuroticism might. That is, team members may be able to put up with this behavior for a short task duration, whereas it may be detrimental to teams over a longer duration. Researchers have sounded the call to examine the possibility of curvilinear relationships (e.g., Webber and Donahue, 2001; Triana et al., 2021; Byron et al., 2023), yet much is left to be learned.

The potential presence of curvilinear relationships also depends on the adopted aggregation approach (Chan, 1998). Kozlowski and Klein (2000) note that two general types of aggregation approaches – how individual-level attributes are aggregated to form a team-level variable – exist. The approaches include the compositional model and the compilational model. The compositional model, as Bell et al., 2011 note, adopts an isomorphic approach to team composition, assuming that individual contributions are equal and thus aggregation techniques reflect this (e.g., sum, average, variance). The compilational model assumes that the formation of a team-level variable is better represented by aggregation approaches that take into account differential contributions (e.g., minimum and maximum scores, weighting techniques).

Moreover, as Stewart (2006) notes regarding team composition, “One line of research examines aggregated characteristics to assess whether the inclusion of individuals with desirable dispositions and abilities improves team performance. A related but somewhat different area of research looks at how heterogeneity of individual characteristics relates to team outcomes” (p.30). This second line of research focuses on diversity. Similarly, how diversity is conceptualized (i.e., as variety, disparity, or separation; Harrison and Klein, 2007) can have an impact on the diversity-performance relationship.

## Work and task design

Work design refers to how tasks and task activities are organized within the team (Handke et al., 2020). There are a number of questions to be examined in terms of the structure and organization of the fluid team environment.

### The impact of team size on fluid teams

Overall, past research reveals a positive team size-performance relationship (Stewart, 2006; Hülsheger et al., 2009; Jin et al., 2017; Carter et al., 2019). Steiner (1972) noted that a team’s size may impact performance in several ways. As a group gets larger, the diversity of its members increases, thus increasing the variety of viewpoints and potentially increasing the number of problem solutions available to the team. However, larger teams do not always perform better than smaller ones. Increasing team size makes coordination requirements more difficult, and as groups increase in size and complexity, more group structure (i.e., role differentiation, etc.) is required to coordinate group activity. It is well established that, while total group performance may increase as the size of the group increases, group member performance (i.e., performance per person) and satisfaction decrease as a function of group size (e.g., Mullen and Baumeister, 1987; Mullen, 1991).

However, as Stewart (2006) notes, “the benefits of a larger team likely depend on the nature of the team and its environment” (p. 33). This rings especially true for fluid teams. Traditional or tenured teams have the luxury of working out difficulties associated with the added complexity brought on by more team members. That is, tenured teams have the time to develop the processes and cognitions (e.g., transactive memory) required to overcome these complexities. Given that fluid teams may not have the opportunity to fully develop these processes and cognitions, or at least not at the level of tenured teams, fluid teams may struggle when more members are added. Research is needed to substantiate this proposition.

### Leadership in fluid teams

Ginnett (1986) noted that leadership is critical to the performance of *ad hoc* teams, and that the leader can set the boundaries for team interaction, clarify aspects of the task that require coordination (both within the team, and with other teams), and foster norms that encourage teamwork. Leadership styles vary and consequently so can their effectiveness. Burke et al. (2006) investigated task-focused leadership (including transactional, initiating structure, and boundary spanning) and person-focused leadership (including transformational, consideration, empowerment, and motivational). They found that all types of leadership benefited team performance. The question becomes, which type of leadership is most efficacious for fluid teams? Given that fluid teams require disparate and distributed team members to rapidly assemble, perform a mission, and then dissemble, shared leadership and boundary spanning may be particularly relevant. Additionally, the moderating effects of leadership style on the composition-performance relation is unclear. That is, do fluid teams composed of certain team members (e.g., all members being domain experts) function better under certain leadership styles? Research is needed to answer these questions.

### The impact of role definition and clarification on fluid team performance

The extant literature has emphasized the importance of role clarity in newly formed teams (Driskell et al., 2017; McLeod et al., 2021). Moreover, Blomqvist and Cook (2018) noted that role clarity “facilitates trust building in such time-constrained situations in which strangers come together relatively quickly to jointly accomplish a task” (p. 7). Research is needed to examine whether greater role clarity and specification enhances team functioning as well as the development of initial trust in fluid teams.

### Pre-briefing activities to support team member orientation

Morgan et al. (1993) noted that initial team member orientation behaviors are focused on understanding the nature of the task itself (taskwork), or to understanding the team-dependent aspects of performance (e.g., teamwork; expectations regarding other team members). Briefings are often designed to address the taskwork component, whereas a team orientation-focused pre-briefing can address the teamwork component, with the goal of orienting new team members to work with one another quickly. This may be particularly relevant for fluid teams in that the opportunity for initial orientation to other team members is compromised by the time-compressed nature of the task. Morgan et al. (1993) incorporated a Pre-Forming stage in their team development model, recognizing that events may occur prior to convening of the team that may address issues of initial orientation. Research is needed to develop and test the value of preparatory pre-briefing procedures (see Driskell et al., 2008) to support initial team member orientation in fluid contexts.

### The use of biographical data to foster trust

Providing relevant biographical data to team members on other team members that they will interact with prior to fluid team assembly may provide one means to foster an initial level of familiarity, common ground, and trust. It is likely that easily discernable surface-level characteristics (e.g., rank, organizational affiliation) provide the primary means of forming initial trust judgments of others in newly formed teams. Several questions arise about the potential use of this approach. First, what types of biographical information (e.g., experience, competencies) are most relevant? What types of biographical information are most easily conveyed? Third, how should this information be conveyed (e.g., the use of biographical “ID” cards)? Finally, to the extent that reliance on surface-level characteristic in forming task expectations can foster stereotypical biases, research is needed on minimizing negative consequences of such categorization.

### Pre-performance team communication

Marks et al. (2001) proposed that “team members interpret their charge within the boundaries of team abilities, resources, and time constraints. This process also includes verbal discussion, to ensure that all members have a shared vision of the team’s purpose and objectives” (p. 365). Capiola et al. (2020) further note that pre-performance communication may provide a means to gain information and initial familiarity with new team members. Although the time-constrained nature of fluid team tasks means that this opportunity is limited, even brief interaction or online chat may provide a degree of familiarization with teammates prior to task execution. Research is needed to investigate the possibility of providing brief team member communications for orientation purposes prior to assembling for task performance.

## Training

There is a robust literature on team training (see Cannon-Bowers et al., 1995; Stagl et al., 2007; Salas et al., 2008) and much of this can be leveraged in supporting fluid team performance. However, whereas team training optimally involves the training of intact teams, fluid teams are typically not available as an intact unit for team training. That is, they do not exist prior to team performance. Research is needed to examine the implications of training for fluid team contexts.

### Can propensity to trust be modified?

Trust is expected to be central to newly formed fluid teams. Propensity to trust refers to the dispositional baseline level of trust that the individual is willing to extend to others (Wildman et al., 2012). Jarvenpaa et al. (1998) discovered that members’ *own propensity to trust* had a significant effect on trust. Moreover, dispositional trust is also predictive of trust before adequate information is known about the referent (Capiola et al., 2020). This raises the question of whether such predispositions can be modified or changed (e.g., through task design or training). Certain predispositions, such as the propensity to trust, may be viewed as less stable than personality traits and malleable enough to be changed through experience or training (see Mohammed and Angell, 2004; Driskell et al., 2006). Research is needed to examine whether the baseline propensity to trust can be modified prior to fluid team formation.

### How can you assist fluid teams to take advantage of knowledge, skill, and ability (KSA) diversity?

The informational diversity–cognitive resource perspective suggests that diversity should provide a greater corpus of KSAs to draw upon, which should enhance team performance (see Driskell et al., under review). The important qualification here is presuming teams can leverage a greater KSA base during task performance (Webber and Donahue, 2001). This qualification is especially germane for fluid teams. That is, the requirement to leverage unique skills is going to be predicated on team members’ knowledge of who has what skills, and members of fluid teams are likely to have more difficulty developing a transactive memory system representing an understanding of who on the team has what knowledge and expertise. Research is needed to explore how KSA diversity can be leveraged to improve team performance.

While diversity can lead to conflict, which can interrupt team process and performance, recent research suggests that training can be leveraged to minimize these disruptions and allow team members to unite through their differences (Davis et al., 2022). Specifically, Davis and colleagues propose integrating training with emotional management to accomplish this objective. More research is warranted, especially given the practical advantages of this approach.

### How do you enhance fluid team resilience?

Composing teams that perform well and composing teams that perform well under stress are distinct tasks (Driskell et al., 2018). For example, certain attributes may become more or less important when teams are required to perform in high demand conditions. Fluid team contexts are often high-demand settings in which the team is formed to address a time-sensitive critical task, and research is needed to identify core attributes that help teams ward off the adverse effects of stress. Fluid teams are unlikely to develop strong interpersonal bonds or the level of social support possible in longer-term interactions, nor are team members likely to have extensive experience in fluid team contexts to draw upon. Future research is needed to investigate resilience factors that are relevant to fluid team performance.

### Development of a fluid team shared mental model

*Shared mental models* (SMMs) reflect a teams’ shared understanding about how to execute a task and how to work effectively (e.g., coordinate efforts) as team members to accomplish that task, and have been shown to be a strong predictor of team performance (DeChurch and Mesmer-Magnus, 2010). However, most research on SMMs has been conducted on traditional teams. It has been suggested that because fluid teams are rapidly-formed teams of strangers, the shared understanding of how to execute tasks (i.e., taskwork mental model) and work as a team (i.e., teamwork mental model) is likely to be more problematic (Driskell et al., 2024). Potential research questions abound: How does a fluid team SMM differ from traditional teams? What are the characteristics of a fluid team mental model? How can a shared teamwork mental model be developed in a fluid team context? Can training or prior experience in fluid teams foster the development of a fluid team SMM? Much research is needed.

## Researching fluid teams

Table 1 provides a summary of key research topics to support fluid team performance.

**Table 1:** Research agenda.

Approach	Proposed Research Topics
Selection/Composition	Impact of surface and deep level cues
	Application of existing team selection inventories
	Distribution of multiple attributes
	Broad vs. lower-level facets
	Curvilinear effect relationships
Work Design	Impact of team size
	Leadership in fluid teams
	Role definition and clarification
	Pre-briefing activities
	Provision of biographical data
	Pre-performance team communications
Training	Trust development
	KSA Diversity
	Building team resilience
	Shared mental models

Finally, we note some of the methodological and measurement challenges in studying fluid teams. As with traditional teams, there are challenges in conducting research in field settings on fluid teams *in situ*. Moreover, there is likely to be little opportunity to gain access to team members prior to team assembly or post-performance. Further, given that many fluid team task contexts involve immediate, time-sensitive and critical tasks (e.g., emergency room procedures, military exercises), it may be a challenge to gain researcher access on even a not-to-interfere or observational basis. On the other hand, the experimental laboratory provides a research setting that may in many ways serve as an approximation of a fluid team context, given that laboratory research participants are often unfamiliar with one another, convene for a specific task, and then disband. Of course, appropriate caution should be exercised in considering the advantages and disadvantages of either research setting.

Researchers should also consider how measurement may differ in fluid versus traditional teams. To the extent that many critical properties of teams emerge over time (e.g., cohesion, trust), these emergent properties differ in short-term fluid teams. Thus, for example, researchers have identified measures of “swift trust” relevant to *ad hoc* teams (Meyerson et al., 1996; Capiola et al., 2020). It is further likely that many measures used in traditional team research can be directly borrowed to measure fluid teams because these measures, overall, assess what a team feels, does, thinks (e.g., team processes and emergent states), and produces. However, consideration of the fluid team context must be taken into consideration by the researcher regarding the use of these measures. For example, construct measures are often multi-dimensional, and in many instances, certain sub-dimensions will be more applicable to fluid teams. Cohesion is one example; most researchers adopt a multi-dimensional conceptualization of cohesion (including the sub-dimensions of interpersonal cohesion, task cohesion, and group pride; see Forsyth, 2021), and for short-term fluid teams, task cohesion may be the most relevant measure. Further research is warranted on adaptation of traditional measures for fluid team contexts.

## Concluding remarks

This article has summarized a research agenda aimed at providing avenues for future research on fluid teams. To date, fluid teams have been understudied, yet their relevance and application in the modern workplace is expanding. Moreover, fluid teams are prevalent in task domains that are high-risk and high-consequence (e.g., military combat units, surgery teams, disaster response teams). Future research should lead to a greater understanding of fluid team performance.

## Data availability statement

The original contributions presented in the study are included in the article/supplementary material, further inquiries can be directed to the corresponding author.

## Author contributions

TD: Writing – original draft, Writing – review & editing. GF: Writing – review & editing. MT: Writing – review & editing. AC: Writing – review & editing. JD: Writing – review & editing.
